# Authors’ reply

**DOI:** 10.4103/0970-2113.80341

**Published:** 2011

**Authors:** Prasanta R. Mohapatra, Naveen Dutt, Sushant Khanduri, Baijayantimala Mishra, Ashok K. Janmeja

**Affiliations:** *Department of Pulmonary Medicine, Government Medical College and Hospital, Chandigarh, India*; 1 *Department of Virology, Postgraduate Institute of Medical Education and Research, Chandigarh, India. E-mail: prmohapatra@hotmail.com*

Sir,

We thank Singh and colleagues for highlighting the weakness of noninvasive ventilator (NIV) in acute respiratory failure due to H1N1 infection.[1] We highly appreciate their comments and are happy to share and debate the controversy in light of the arguments. Awareness of the limitations and advantages of NIV is crucial for patient management. Benefits of such a service need to be balanced against increased costs in a resource-poor setting.

We have already discussed the use of NIV in a resource-poor setting or when invasive ventilator is unavailable during a high demand situation, particularly in selected patients with respiratory failure.[2] The same guidelines[3] quoted above also mention that “NIV may be considered to prevent further deterioration and need for intubation in patients with mild to moderate hypercapnic acute respiratory failure due to H1N1 infection" and also suggested the methods of limiting droplet dispersion and disease transmission during NIV in patients with H1N1 infection. Again the laboratory confirmation of H1N1 may take days. We had come across the situation where the need of mechanical ventilators was out of proportion to the resources available in the hospital during peak incidences of H1N1. While awaiting confirmation, it is justified to use NIV in the scenario of nonavailability of invasive ventilator.

Singh and colleagues mentioned that “only a few patients with H1N1-related respiratory failure who seem to benefit from NIV alone.” In this regard, we still have limited data to comment or conclude. However, most of the few cases published so far were effectively managed with NIV.

The second point raised by them has also been partially answered above. We should not compare the nosocomial outbreaks of severe acute respiratory syndrome (SARS) with that of H1N1 infection due to NIV. The virulence and secondary attack rate of SARS were much higher. The basic reproduction number (the average number of individuals whom each infected individual will infect, in a nonimmune population) for the 2009 novel H1N1 is estimated to be 1.75 (95% confidence interval 1.64–1.88).[4] SARS was an epidemic and this was a pandemic. There was community spread of H1N1virus and subsequent development of self immunity and finally on 10th August 2010 WHO had declared the end of pandemic.

Every procedure has some acceptable risks. Even the mechanical ventilators are not free from the aerosol transmission. Clinical procedures required for invasive ventilation (eg, endotracheal intubation, cardiopulmonary resuscitation, and receipt of high-flow oxygen) can generate a large amount of respiratory aerosols.[5]

Nosocomial outbreak of influenza in an acute ward setting can be temporally related to the use of an aerosol-generating device. This occurs together with an imbalanced indoor airflow; and the spatial distribution of cases in relation to the direction of airflow and aerosol dispersal pattern. We must deliver best care to the patient within the available resources at our setup; we must find better ways to ensure control measures, such as avoiding aerosol generation and improving ward ventilation design, which warrant consideration to prevent all the types of nosocomial outbreaks.
